# Sustained Contraction in Vascular Smooth Muscle by Activation of L-type Ca^2+^ Channels Does Not Involve Ca^2+^ Sensitization or Caldesmon

**DOI:** 10.3389/fphar.2016.00516

**Published:** 2016-12-26

**Authors:** Hillevi K. Ets, Chun Y. Seow, Robert S. Moreland

**Affiliations:** ^1^Department of Pharmacology and Physiology, Drexel University College of Medicine, PhiladelphiaPA, USA; ^2^Department of Pathology and Laboratory Medicine, University of British Columbia, VancouverBC, Canada; ^3^Department of Pathology and Laboratory Medicine, Drexel University College of Medicine, PhiladelphiaPA, USA

**Keywords:** carotid artery, Bay K8644, nifedipine, myosin light chain kinase, protein kinase C, MAP kinase, Rho kinase

## Abstract

Vascular smooth muscle (VSM) is unique in its ability to maintain an intrinsic level of contractile force, known as tone. Vascular tone is believed to arise from the constitutive activity of membrane-bound L-type Ca^2+^ channels (LTCC). This study used a pharmacological agonist of LTCC, Bay K8644, to elicit a sustained, sub-maximal contraction in VSM that mimics tone. Downstream signaling was investigated in order to determine what molecules are responsible for tone. Medial strips of swine carotid artery were stimulated with 100 nM Bay K8644 to induce a sustained level of force. Force and phosphorylation levels of myosin light chain (MLC), MAP kinase, MYPT1, CPI-17, and caldesmon were measured during Bay K8644 stimulation in the presence and absence of nifedipine, ML-7, U0126, bisindolylmaleimide (Bis), and H-1152. Nifedipine and ML-7 inhibited force and MLC phosphorylation in response to Bay K8644. Inhibition of Rho kinase (H-1152) but not PKC (Bis) inhibited Bay K8644 induced force. U0126 significantly increased Bay K8644-dependent force with no effect on MLC phosphorylation. Neither CPI-17 nor caldesmon phosphorylation were increased during the maintenance of sustained force. Our results suggest that force due to the influx of calcium through LTCCs is partially MLC phosphorylation-dependent but does not involve PKC or caldesmon. Interestingly, inhibition of MLC kinase (MLCK) and PKC significantly increased MAP kinase phosphorylation suggesting that MLCK and PKC may directly or indirectly inhibit MAP kinase activity during prolonged contractions induced by Bay K8544.

## Introduction

Vascular smooth muscle (VSM) contraction is initiated by an increase in intracellular Ca^2+^ via influx through plasma membrane ion channels or release from the sarcoplasmic reticulum (Somlyo and Somlyo, 1968). Once in the cytoplasm, Ca^2+^ binds with calmodulin in order to activate myosin light chain (MLC) kinase. This leads to phosphorylation of the regulatory MLC at Ser^19^, interaction between actin and myosin filaments, and contraction (Conti and Adelstein, 1981; Dillon et al., 1981). While the influx of Ca^2+^ and phosphorylation of MLC are required for the initiation of smooth muscle contraction, it is not clearly understood how force is sustained over time. There appears to be a complex, highly nuanced intracellular signaling network that can finely tune the level of maintained force in order to meet the ever-changing circulatory demands of an organism (Horowitz et al., 1996; Davis and Hill, 1999).

Vascular smooth muscle contractile force can be modulated by Ca^2+^ sensitization or thin filament disinhibition. Ca^2+^ sensitization involves inhibition of the MLC phosphatase such that the cell can maintain levels of MLC phosphorylation without additional cytosolic Ca^2+^ or activation of MLC kinase. Rho kinase (ROCK), activated by GTP-bound RhoA, phosphorylates and inhibits the myosin-binding subunit of the MLC phosphatase, a protein known as MYPT1 (Kimura et al., 1996). Additionally, protein kinase C (PKC) phosphorylates the 17 kDa protein kinase C-potentiated myosin phosphatase inhibitor (CPI-17) which, when phosphorylated, binds to and inhibits the MLC phosphatase (Eto et al., 1995). Thin filament disinhibition involves phosphorylation of caldesmon that relieves inhibition of myosin ATPase. The involvement of either of these modulatory pathways is not entirely understood but they are thought to contribute to VSM tone.

Tone is a variable amount of VSM contractile force that exists in the presence of sub-maximal stimulation (Folkow, 1989). A feature of tone is that the intracellular Ca^2+^ concentration ([Ca^2+^]_i_) is higher than during a relaxed state but significantly lower than during a stimulated contractile event (Rubart et al., 1996). This small amount of Ca^2+^ in the cytoplasm is thought to be maintained by a balance of Ca^2+^ influx through calcium channels and efflux via Ca^2+^ATPases and Na^+^/Ca^2+^ exchangers in the plasma membrane (Nazer and van Breemen, 1998; Thornloe and Nelson, 2005). The L-type Ca^2+^ channel (LTCC) appears to be particularly important in maintaining VSM tone (Moosmang et al., 2003; Cobine et al., 2007).

LTCC (also known as voltage-gated calcium channels, Cav_1.2_ channels, and dihydropyridine receptors) can respond to small changes in membrane potential and do not require an action potential to be opened (Fleischmann et al., 1994; Catterall, 2000; Navedo et al., 2005). This allows LTCC to create a “calcium window” that provides a small, sustained influx of Ca^2+^ into the cell that can activate the contractile mechanism (Gollasch et al., 1998; Fransen et al., 2012). Experimental evidence has demonstrated a role for LTCC in VSM tone. A smooth muscle-specific knockout of the gene for LTCC in mice resulted in animals with reduced mean arterial pressure (Moosmang et al., 2003). Another study showed that LTCC are upregulated in genetic rodent models of hypertension (Sonkusare et al., 2006). Human patients with an inherited form of hypertension also exhibit increased expression of LTCC in their vasculature (Pesic et al., 2004). Aberrations in the number or function of LTCC appear to cause pathologic states of hypo- or hypertension (Navedo et al., 2010), but how does this affect intracellular signaling pathways?

The goal of this study was to use a pharmacological agonist of LTCC, Bay K8644, to elicit a sustained, sub-maximal contraction in VSM in order to study the effect on downstream signaling. Inhibitors against proteins known or suspected to be involved with modulation of force were used and the effects on force and intracellular signaling were observed. The results were in many ways unexpected and provided novel insight into what mechanisms sustain force in response to increased LTCC opening.

## Materials and Methods

### Preparation of VSM Tissue

Swine carotid arteries were transported from a local slaughterhouse to the laboratory in ice-cold physiological salt solution (PSS) that contained all ingredients listed below except glucose. Arteries were immediately cleaned of excess connective tissue and fat, then stored in glucose-containing PSS composed of (in mM): 140 NaCl, 4.7 KCl, 1.2 MgSO_4_, 1.6 CaCl_2_, 1.2 Na_2_HPO_4_, 2 MOPS (pH 7.4), 5 D-glucose, and 0.02 EDTA at 4°C for no longer than 4 days. Previous studies have shown there is no loss of viability or contractile force with this storage (Fulginiti et al., 1993; Katoch and Moreland, 1995; Smolock et al., 2006). Prior to experimentation, adventitial and intimal layers were carefully removed in order to obtain a strip composed primarily of medial smooth muscle. Circumferential strips were cut (2 mm ×∼6 mm) and placed in PSS at 4°C until they were mounted for measurement of isometric force.

### Isometric Force Recordings

Circumferential strips of VSM were mounted in double-walled glass muscle baths containing PSS bubbled with 100% oxygen and maintained at 37°C. Tissue strips were stretched to an initial tension of 5 grams and allowed to equilibrate and stress-relax to a baseline of approximately 1 g of passive tension; this passive force has been previously shown to set the tissues at a length that approximates L_o_, a length within the length-range for optimal active force development (Herlihy and Murphy, 1973). Following equilibration, tissues were contracted with 110 mM KCl-containing PSS (equimolar substitution for NaCl) and relaxed with regular PSS. This contraction-relaxation cycle was repeated four times until a stable maximum force value was achieved. Isometric force was detected by Grass FT.03 force transducers and recorded using LabChart software (AD Instruments, Colorado Springs, CO, USA). Tissues were treated with one of the following inhibitors for 20 min: 1 μM nifedipine (LTCC inhibitor), 10 μM ML-7 (MLC kinase inhibitor), 3 μM bisindolylmaleimide I (Bis; PKC inhibitor; henceforth referred to as Bis), 10 μM U0126 (MAP kinase kinase inhibitor), or 1 μM H-1152 (Rho kinase inhibitor). Following treatment with inhibitors 100 nM of the LTCC agonist Bay K8644 was added for 120 min. Measurements of protein phosphorylation were all carried out in muscle tissues frozen at the 120 min time point. Because LTCC are voltage-gated, experiments were conducted in PSS containing 15 mM KCl, rather than the typical 4.7 mM KCl. Preliminary studies showed that 15 mM KCl-containing PSS alone did not produce an increase in force. As shown in several studies, the increased concentration of KCl created a sufficient change in membrane voltage such that LTCC could be activated by Bay K8644 (Gopalakrishnan et al., 1985; Alonso et al., 1989; Navedo et al., 2005). At the conclusion of the experiment, tissues were rapidly frozen in a slurry of 6% trichloroacetic acid/acetone/10 mM dithiothreitol and dry ice at the 120 min time point after stimulation.

### Processing of Tissue Samples

Frozen VSM tissue strips were slowly thawed to room temperature and then rinsed in 100% acetone to remove residual traces of trichloroacetic acid. They were then air dried before being pulverized in an ice-cold buffer solution of 1% SDS, 10% glycerol, 50 mM Tris HCl (pH 6.8), 10 mM dithiothreitol, and Protease Inhibitor Cocktail (Sigma, St. Louis, MO, USA) using glass-glass homogenizers. Samples were stored at –80°C until subjected to SDS-polyacrylamide gel electrophoresis (PAGE). Protein content of homogenized samples was determined using a Lowry protein assay.

### SDS-PAGE and Western Blotting

For detection of CPI-17 and MAP kinase phosphorylation, 20 μg of each sample was loaded onto a 14% polyacrylamide separating gel with a 4% stacking gel. For detection of MYPT1 and caldesmon phosphorylation, 20 μg of each sample was loaded onto an 8% polyacrylamide separating gel with a 4% stacking gel. Gel electrophoresis was carried out at 200 V at room temperature for 1 h or until the dye front reached the bottom of the gel. Proteins were then transferred to a nitrocellulose membrane at 100 V for 1 h in a chilled buffer tank. Membranes were blocked in a 1:1 solution of Odyssey Blocking Buffer (Licor, Lincoln, NE, USA) and phosphate buffered saline (PBS, pH 7.4) at room temperature for 1 h then incubated in primary antibodies diluted in a 1:1 solution of Odyssey Blocking Buffer and PBS overnight at 4°C. Membranes were washed in PBS with 0.1% Tween five times for 5 min each. Fluorescent secondary antibodies (goat anti-mouse 800CW and goat anti-rabbit 680LT, Licor, Lincoln, NE, USA) were used at a 1:10,000 dilution in a 1:1 solution of Odyssey Blocking Buffer and PBS. Membranes were incubated in secondary antibody at room temperature for 45 min followed by five washes in PBS with 0.1% Tween for 5 min each.

Membranes were imaged using a Licor FC camera system and optical densitometry of the bands was determined using Image Studio software (Licor, Lincoln, NE).

### Urea/Glycerol-PAGE

Thawed and dehydrated tissues were placed in sample buffer containing 8 M urea, 18.5 mM Tris (pH 8.6), 20.4 mM glycine, 0.5 M dithiothreitol, 0.05% saturated sucrose, 0.2% bromophenol blue, 0.4 M EDTA, and Protease Inhibitor Cocktail (Sigma, St. Louis, MO, USA). Samples were gently rocked at room temperature for approximately 2 h during which the MLC were solubilized, then stored at 4°C until subjected to gel electrophoresis. A gel was prepared containing 18.5 % glycerol, 30% acrylamide with bisacrylamide (29:1), Tris (pH 8.6), glycine, and water. The gel was subjected to pre-electrophoresis at 400 V for 1 h. Samples were then loaded onto the gel, subjected to electrophoresis at 400 V for 90 min, then transferred to a nitrocellulose membrane at 25 V for 1 h. Western blotting was carried out as described above for SDS-PAGE.

### Chemicals and Antibodies

Drugs and inhibitors were dissolved either in deionized water or dimethyl sulfoxide (DMSO) according to manufacturers’ instructions. Bay K8644 and ML-7 were obtained from Tocris (Bristol, UK). Nifedipine was obtained from Sigma (St. Louis, MO, USA). H-1152, U0126, and Bis, were ordered from Calbiochem (Billerica, MA, USA). The following antibodies were used for Western blotting: p42/44 MAPK (Millipore, Billerica, MA, USA), phosphorylated p42/44 MAPK^Thr185/Tyr187^ (Promega, Madison, WI), MLC (Sigma, St Louis, MO, USA), phosphorylated MLC^Ser19^ (Cell Signaling, Danvers, MA, USA), CPI-17 (Santa Cruz, Santa Cruz, CA, USA), phosphorylated CPI-17^Thr38^ (Santa Cruz, Santa Cruz, CA, USA), MYPT1 (Covance, Conshohocken, PA, USA), phosphorylated MYPT1^Thr850^ (Millipore, Billerica, MA, USA), caldesmon (Sigma, St Louis, MO, USA), and phosphorylated caldesmon^Ser789^ (Millipore, Billerica, MA, USA). Electrophoretic reagents were purchased from Bio-Rad Laboratories (Hercules, CA, USA). All other chemicals were analytical grade or better and purchased from Fisher Scientific (Pittsburgh, PA, USA).

### Statistical Analysis

Results are represented as mean ± SEM with n representing the number of arteries. Statistical analysis was performed using One-way ANOVA with the Holm–Sidak Method of comparing experimental groups to Bay K8644-stimulated tissue. Time course of force development was analyzed with a One Way Repeated Measures ANOVA. A *p*-value less than 0.05 was considered significant.

## Results

### Bay K8644-Stimulated Force

**FIGURE 1:**
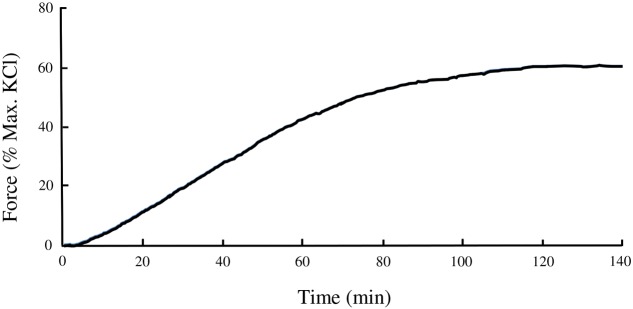
**Time course of isometric contraction of swine carotid smooth muscle stimulated by 100 nM of Bay K8644.** Measurements of plateau force and protein phosphorylation were done at the 120 min time point.

**FIGURE 2:**
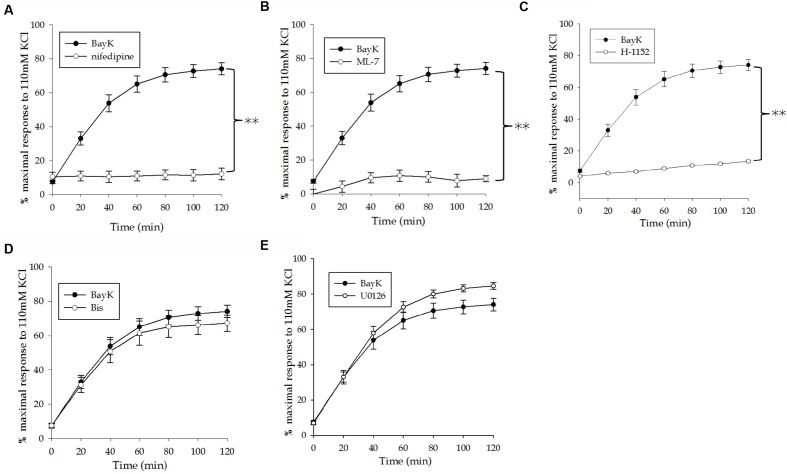
**Effect of pharmacological inhibitors on Bay K8644-stimulated force.** **(A)** Effect of the LTCC inhibitor nifedipine (1 μM) on a 100 nM Bay K8644 induced contraction of the swine carotid artery. **(B)** Effect of the MLC kinase inhibitor ML-7 (10 μM) on a 100 nM Bay K8644 induced contraction. **(C)** Effect of the Rho kinase inhibitor H-1152 (1 μM) on a 100 nM Bay K8644 induced contraction. **(D)** Effect of the pan-PKC inhibitor Bis (3 μM) on a 100 nM Bay K8644 induced contraction. **(E)** Effect of the MAP kinase kinase inhibitor U0126 (10 μM) on a 100 nM Bay K8644 induced contraction. Inhibiting LTCC, MLC kinase, and Rho kinase significantly prevented force development. Inhibiting PKC had no appreciable effect on force. Inhibiting MAP kinase kinase increased steady state force but did not affect the rate of rise. Values shown are means ± SEM as a percent of the maximal force in response to 110 mM KCl-PSS. *n* = 10–20, each tissue strip was taken from a different artery. One way repeated measures ANOVA was performed to detect differences between Bay K8644 induced contractions alone or in the presence of the various pharmacological inhibitors. ^∗∗^*p* < 0.001 as compared to Bay K8644 in the absence of inhibitor.

The swine carotid artery exhibits little spontaneous tone therefore a pharmacological approach was used to mimic tone in this tissue. A dose-response curve of VSM contraction to the LTCC agonist Bay K8644 (30, 100, 300, 1, 3, and 10 μM) was used to determine the concentration(s) that produced sub-maximal force (data not shown). A time course experiment revealed that Bay K8644-induced force increased steadily for the first 60 min and very slowly over the next 60 min. The optimal concentration for sustained, sub-maximal force was determined to be 100 nM. The magnitude of force development was normalized to the maximum force produced by a tissue strip in response to 110 mM KCl PSS. Bay K8644-induced force was approximately 60–70% of maximal KCl-stimulated force, as shown in **Figure 1**. To study the potential role of various signaling proteins in VSM contraction, inhibitors against LTCC (nifedipine), MLC kinase (ML-7), PKC (Bis), MAP kinase kinase (U0126), and Rho kinase (H-1152) were added 20 min prior to stimulation with Bay K8644. Pre-treatment with nifedipine (**Figure 2A**) or ML-7 (**Figure 2B**) prevented Bay K8644-induced force development. Inhibition of ROCK by H-1152 (**Figure 2C**) also prevented force development suggesting that ROCK activity is required for initiation of membrane depolarization-induced force. Inhibition of PKC with Bis did not significantly alter Bay K8644-stimulated force (**Figure 2D**) suggesting that PKC activity is not required for force initiation or maintenance in response to opening LTCC. Pre-treatment with the MAP kinase kinase inhibitor U0126 produced greater sustained force than Bay K8644 stimulation without inhibitor (**Figure 2E**) however, the rate of rise was the same (i.e., no leftward shift of the curve). The finding that inhibition of MAP kinase kinase increases sustained force suggests that MAP kinase may have an inhibitory role in contraction, perhaps to keep the tissue from unregulated maximal contractions. As can be seen at zero time in the panels in **Figure 2**, basal, unstimulated force was approximately 5–10% of the maximum response to 110 mM KCl.

**FIGURE 3:**
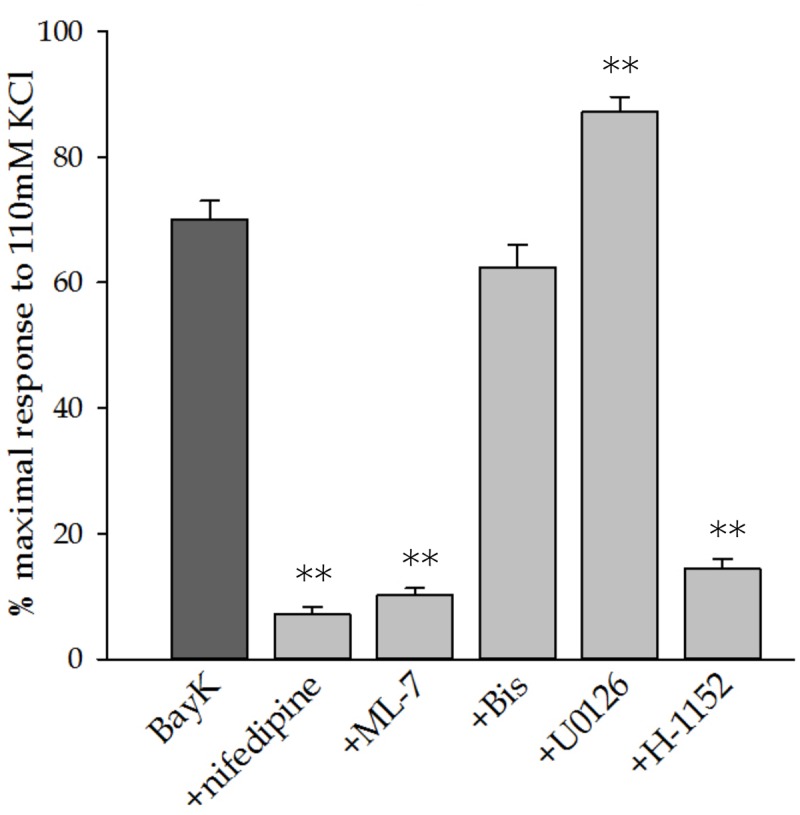
**Steady state force in response to 100 nM Bay K8644 after 120 min of stimulation.** Bay K8644-stimulated force at the termination of the experiment (120 min) was compared to a maximal contraction in response to 110 mM KCl-PSS. Nifedipine (LTCC inhibitor), ML-7 (MLC kinase inhibitor), and H-1152 (Rho kinase inhibitor) significantly decreased steady-state force. Bis (PKC inhibitor) did not affect force. U0126 (MAP kinase kinase inhibitor) significantly increased steady-state force. Addition of U0126 did not affect the rate of force development, only the magnitude of steady-state force. Values shown are the means ± SE and presented as a percent of the maximal response to 110 mM KCL-PSS. *n* = 10–20, each tissue strip was taken from a different artery. One way ANOVA with multiple comparisons against Bay K8644 contractions in the absence of pharmacological inhibitors (Holm–Sidak Method) was performed to determine significance. ^∗∗^*p* < 0.001 as compared to Bay K8644 stimulation without inhibitor.

**Figure 3** shows the force developed in response to Bay K8644 alone and in the presence of the various inhibitors at the final time point, 120 min after Bay K8644 stimulation. Consistent with the time course data presented in **Figure 2**, inhibition of LTCC, MLC kinase, and ROCK inhibited Bay K8644-induced force whereas inhibition of MAP kinase kinase increased force in response to Bay K8644. The statistical analysis comparing force at this single time point found the increase during MAP kinase kinase inhibition to be significant.

### Myosin Light Chain Phosphorylation

**FIGURE 4:**
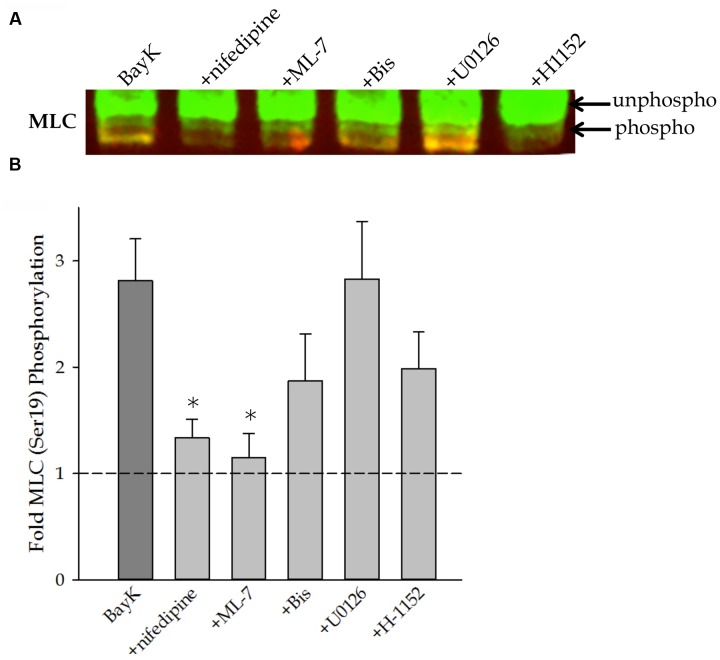
**MLC^Ser19^ phosphorylation measured by urea/glycerol-PAGE in the swine carotid artery in response to 100 nM Bay K8644 in the absence and presence of pharmacological inhibitors.** **(A)** Representative urea/glycerol-PAGE of phosphorylated and unphosphorylated MLC after 120 min of stimulation with 100 nM Bay K8644 in the presence and absence of pharmacological inhibitors. Note the mobility shift between the phosphorylated and unphosphorylated MLC. The top arrow points to the bands of unphosphorylated MLC, the bands at the level of the bottom arrow and below are phosphorylated MLC. The baseline condition (unstimulated without any inhibitor) is not shown. **(B)** Quantitative results of several blots as shown in **(A)**. Bay K8644 without inhibitor increased MLC phosphorylation. Nifedipine (1 μM) and ML-7 (10 μM) abolished Bay K8644-dependent increases in MLC phosphorylation. Bis (3 μM) and H-1152 (1 μM) decreased MLC phosphorylation to some extent but the decrease was not significant. U0126 (10 μM) did not alter Bay K8644-induced increases in levels of MLC phosphorylation. For each condition fractional phosphorylation was obtained [i.e., the volume density of bands of phosphorylated/(phosphorylated + unphosphorylated)], and each of the fractional phosphorylation was then divided by the fractional phosphorylation under baseline condition to obtain the fold increase over the baseline. Values shown are the means ± SE and presented as a fold increase above basal MLC phosphorylation (unstimulated and without inhibitor). *n* = 8–14, each tissue strip was taken from a different artery. One way ANOVA with multiple comparisons against Bay K 8644 contractions in the absence of pharmacological inhibitors (Holm–Sidak Method) was performed to determine significance. ^∗^*p* < 0.05 as compared to Bay K8644 stimulation without inhibitor.

Myosin light chain (MLC) phosphorylation at Ser^19^ was assessed by urea/glycerol-PAGE. Urea/glycerol-PAGE uses a high concentration of urea in the sample buffer to solubilize MLC and separate them from the larger heavy chain molecules. The glycerol gel is suitable for separating different phosphorylation states of the same protein. Phosphorylated and non-phosphorylated MLC have different electrophoretic mobilities within the gel and appear as discrete bands. A representative Western blot is shown in **Figure 4A**. **Figure 4B** shows quantitative results of several such Western blots and demonstrates that Bay K8644 significantly increased Ser^19^ MLC phosphorylation. Nifedipine and ML-7 treatment prevented any increase in Ser^19^ MLC phosphorylation above basal levels, as expected. The MAP kinase kinase inhibitor U0126 had no effect on Bay K8644-stimulated Ser^19^ MLC phosphorylation levels in contrast to significantly increased Bay K8644-induced force. The PKC and ROCK inhibitors (Bis and H-1152, respectively) appeared to reduce the Bay K8644-stimulated MLC phosphorylation to some extent, but failed to bring the phosphorylation level back to the baseline. Due to the large variation in data, it is difficult to draw any meaningful correlation between the phosphorylation level and force production.

### p42/44 MAP Kinase Phosphorylation

**FIGURE 5:**
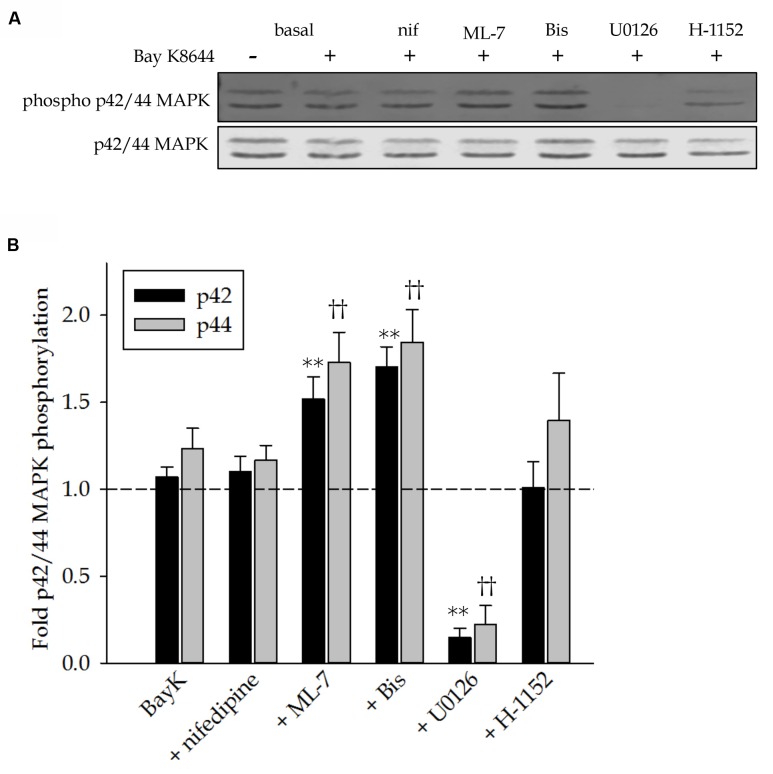
**p42 and 44 MAP kinase phosphorylation levels in response to Bay K8644 in the absence and presence of pharmacological inhibitors in the swine carotid artery.** **(A)** Representative Western blot of p42 and p44 MAP kinase phosphorylation levels in response to 120 min of stimulation with 100 nM Bay K8644 in absence and presence of nifedipine (1 μM, inhibitor of LTCC), ML-7 (10 μM, inhibitor of MLC kinase), Bis (3 μM, inhibitor of PKC), U0126 (10 μM, inhibitor of MAP kinase kinase), and H-1152 (1 μM, inhibitor of ROCK). **(B)** Quantified results of several blots as shown in **(A)**. Dashed line represents basal, unstimulated MAP kinase phosphorylation levels for comparison. 120 min of stimulation with 100 nM Bay did not increase MAP kinase phosphorylation levels. Nifedipine neither increased nor decreased MAP kinase phosphorylation levels during the Bay K8644 stimulation. ML-7 significantly increased both p42 and p44 MAP kinase phosphorylation levels. Similarly, Bis increased phosphorylation of both isoforms of MAP kinase. U0126 abolished basal levels of p42 and p44 MAP kinase. H-1152 had no effect on MAP kinase phosphorylation during Bay K8644 stimulation. Values shown are means ± SEM and are presented as fold change above or below basal values. *n* = 8–14, each tissue was taken from a different artery. One way ANOVA with multiple comparisons (Holm–Sidak Method) was used to determine against values obtained during Bay K8644 stimulated in the absence of inhibitors. ^∗∗^*p* < 0.01 p42 MAP kinase phosphorylation levels as compared to those during Bay K8644 stimulation without inhibitor. ^††^*p* < 0.01 p44 MAP kinase phosphorylation levels as compared to those during Bay K8644 without inhibitor.

Activity of p42/44 MAP kinase was assessed by comparing phosphorylation levels to basal unstimulated control values. **Figure 5A** shows a representative Western blot of MAP kinase phosphorylation in response to Bay K8644 without inhibitor and in the presence of nifedipine, ML-7, Bis, U0126 or H-1152. Quantitation of several such blots is shown in **Figure 5B**. Stimulation of the tissues with Bay K8644 did not significantly increase MAP kinase phosphorylation above basal levels, represented by the dashed line. The effectiveness of U0126 to inhibit the MAP kinase kinase and therefore MAP kinase phosphorylation is demonstrated by the nearly complete abolishment of MAP kinase phosphorylation. Although Bay K8644 alone did not increase MAP kinase phosphorylation, the addition of either Bis to inhibit PKC or the addition of ML-7 to inhibit MLC kinase significantly increased MAP kinase phosphorylation levels. This was unexpected as PKC is thought to activate MAP kinase in this tissue and MLC kinase has no known role in the activation of MAP kinase. Inhibition of LTCC with nifedipine had no effect on MAP kinase phosphorylation.

### MYPT1 Phosphorylation

**FIGURE 6:**
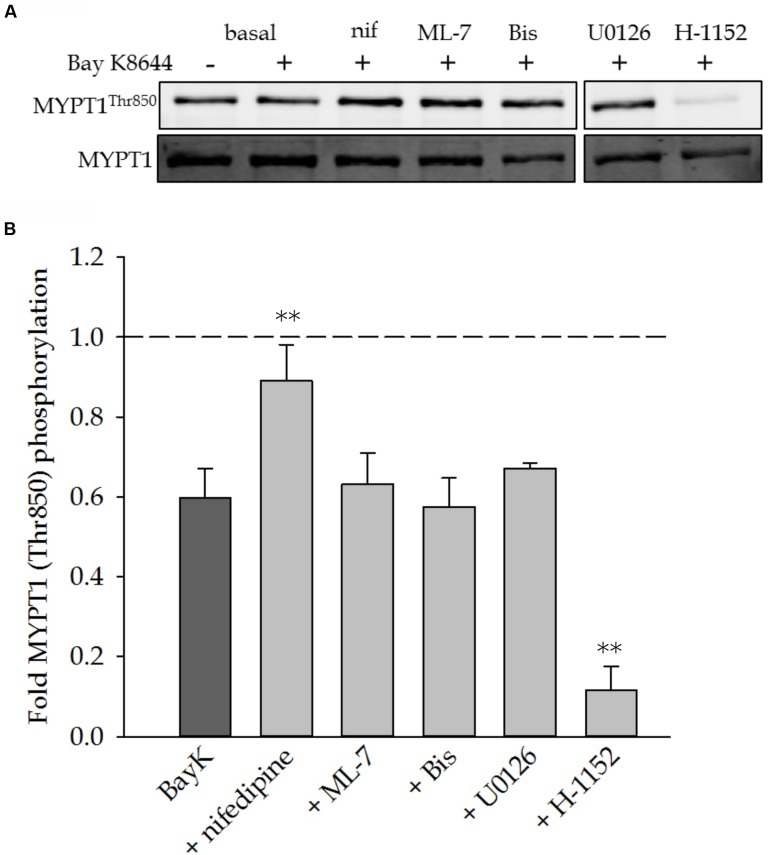
**MYPT1^Thr850^ phosphorylation levels after 120 min of stimulation with Bay K8644 in the absence or presence of pharmacological inhibitors in the swine carotid artery.** **(A)** Representative Western blot of MYPT1^Thr850^ in response to 100 nM Bay K8644 in the absence or presence of nifedipine (1 μM, inhibitor of LTCC), ML-7 (10 μM, inhibitor of MLC kinase), Bis (3 μM, inhibitor of PKC), U0126 (10 μM, inhibitor of MAP kinase kinase), and H-1152 (1 μM, inhibitor of ROCK). **(B)** Quantified results of several blots as shown in **(A)**. Bay K8644 decreased MYPT1^Thr850^ phosphorylation levels from basal, unstimulated levels (dashed line). This decrease was reversed or prevented by the addition of nifedipine. ML-7, Bis, and U0126 had no affect on the Bay K8644-dependent decrease in MYPT1^Thr850^ phosphorylation levels. H-1152 significantly decreased MYPT1^Thr850^ phosphorylation levels as compared to the decrease induced by Bay K8644 stimulation. Values shown are the means ± SEM and presented as a fold change from basal values. *n* = 8–14 with each tissue taken from a different artery. One-way ANOVA with multiple comparisons versus Bay K8644 treatment without inhibitor (Holm–Sidak Method) was performed to determine significance. ^∗∗^*p* < 0.001 as compared to Bay K8644 treated tissues.

To determine the possible involvement of the MLC phosphatase in sustaining VSM force, we measured phosphorylation of the myosin binding subunit, MYPT1. Phosphorylation of MYPT1 inhibits MLC phosphatase activity and allows for maintained MLC phosphorylation and force. **Figure 6A** shows a representative Western blot of MYPT1 phosphorylation in response to stimulation with Bay K8644 without inhibitor and in the presence of nifedipine, ML-7, Bis, U0126, or H-1152. **Figure 6B** shows the quantitation of several such blots. The level of MYPT1 phosphorylation decreased from basal levels (dashed line) in response to Bay K8644 stimulation without inhibitor. The inclusion of ML-7, Bis, or U0126 had no effect on MYPT1 phosphorylation as compared to Bay K8644 without inhibitor. The ROCK inhibitor H-1152 abolished MYPT1 phosphorylation as expected as ROCK is the primary kinase that catalyzes MYPT1 phosphorylation. The level of MYPT1 phosphorylation in the presence of nifedipine was the same as that under baseline conditions (no inhibitor and unstimulated, dashed line in **Figure 6**), suggesting that MYPT1 dephosphorylation may be Ca^2+^-dependent consistent with the decrease in MYPT1 phosphorylation in response to Bay K8644 without inhibitor.

### CPI-17 and Caldesmon Phosphorylation

**FIGURE 7:**
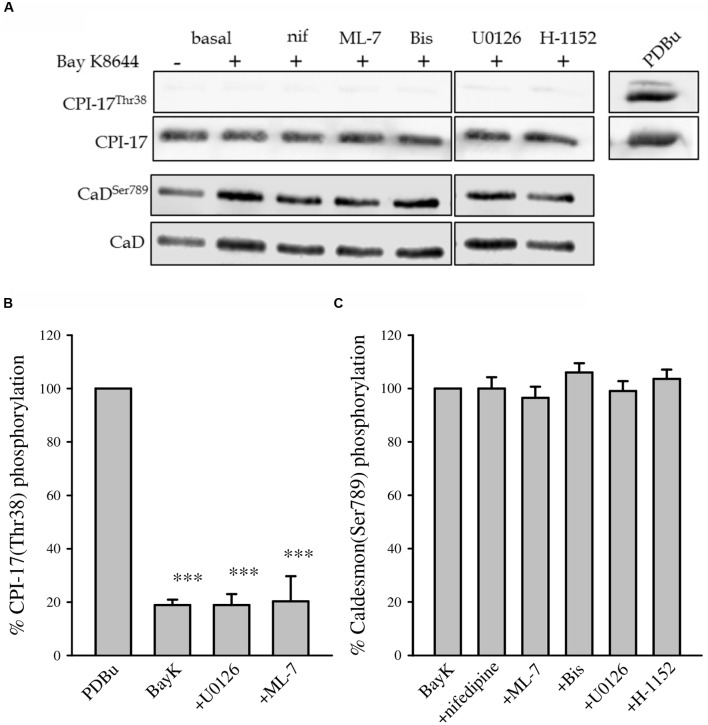
**CPI-17^Thr38^ and caldesmon ^Ser789^ phosphorylation levels after 120 min of stimulation with 100 nM Bay K8644 in the swine carotid artery.** **(A)** Representative Western blots showing CPI-17^Thr38^ and caldesmon^Ser789^ phosphorylation levels during Bay K8644 stimulation in the absence or presence of nifedipine (1 μM, inhibitor of LTCC), ML-7 (10 μM, inhibitor of MLC kinase), Bis (3 μM, inhibitor of PKC), U0126 (10 μM, inhibitor of MAP kinase kinase), and H-1152 (1 μM, inhibitor of ROCK). Phorbol 12,13-dibutyrate (PDBu) was used as a positive control for the measurement of CPI-17^Thr38^. **(B)** Quantified results of several blots as shown in **(A)**. Bay K 8644 stimulation did not increase phosphorylation values of either CPI-17^Thr38^. None of the pharmacological inhibitors increased or decreased CPI-17^Thr38^ phosphorylation level compared with that of BayK. Only three conditions (BayK, U0126, and ML-7) are plotted against the positive control (PDBu), they are all significantly different from the positive control. ^∗∗∗^*p* < 0.001. **(C)** Quantified results of several blots as shown in **(A)**. Bay K 8644 stimulation did not increase phosphorylation values of caldesmon^Ser789^. None of the pharmacological inhibitors increased or decreased caldesmon^Ser789^ phosphorylation level compared with that induced by Bay K8644 stimulation.

The MLC phosphatase inhibitor CPI-17 is phosphorylated by PKC. CPI-17 phosphorylation is usually increased during contraction of VSM as it is one mechanism that increases myofilament Ca^2+^ sensitivity. Caldesmon is a thin filament-associated inhibitory protein that is phosphorylated by MAP kinase and potentially PKC which reverses its’ inhibitory activity (Barany et al., 1992; Childs and Mak, 1993; Wang, 2001). **Figure 7** shows CPI-17 and caldesmon phosphorylation in response to Bay K8644 without inhibitor and in the presence of nifedipine, ML-7, Bis, U0126, or H-1152. The lack of CPI-17 phosphorylation during Bay K8644 stimulation with or without inhibitors suggests that PKC is not activated or that this particular pathway is not involved in the observed increase in Bay K8644-stimulated force and MLC phosphorylation. This agrees with our earlier observation that inhibition of PKC did not affect Bay K8644-induced force development (**Figure 2D**) or MLC phosphorylation (**Figure 4B**). Phorbol-dibutyrate (PDBu) was used as a positive control for CPI-17 phosphorylation as it directly activates PKC. PDBu produced a significant increase in CPI-17 phosphorylation demonstrating that the total lack of CPI-17 phosphorylation was not an artifact due to electrophoresis or Western blotting.

Bay K8644 stimulation alone or in the presence of the various kinase inhibitors had no effect on phosphorylation of the thin filament-associated protein caldesmon at Ser^789^ (**Figure 7**). The level of phosphorylation was the same in basal unstimulated tissues as it was in Bay K8644-stimulated tissues with or without inhibitors. This suggests that thin filament disinhibition by caldesmon phosphorylation is not involved in sustained LTCC-mediated force in VSM. Inhibition of MAP kinase with U0126 had no effect on caldesmon phosphorylation.

## Discussion

The purpose of this study was to investigate intracellular signaling involved in sustained, sub-maximal force induced by the LTCC agonist, Bay K8644. Pharmacological inhibitors of LTCC, MLC kinase, MAP kinase, PKC, and ROCK were used in order to determine their involvement in sustained force.

Before we discuss the effects of the various inhibitors used in this study, it should be pointed out that non-specificity of pharmacological inhibitors is always a potential problem and the accuracy of our data interpretation to a large extent depends on how specific an inhibitor inhibits its intended target and what other non-intended targets are affected. The inhibitors we have chosen are commonly used in pharmacological studies and according to the literature their specific is relatively high. But none of the inhibitors used has only a single target. ML-7 is more specific than its parent form ML-9 in inhibiting MLC kinase (MLCK), however, it is able to inhibit other enzymes. It has effects on cAMP-dependent protein kinases, PKC, and calcium phosphodiesterase, although the binding affinity of ML-7 for smooth muscle MLCK is ∼100 times higher than that for the other enzymes (Saitoh et al., 1987). Although H-1152 is more specific than its parent form HA-1077 and another Rho-kinase inhibitor Y-27632, with a half maximal inhibitory concentration (IC_50_) that is ∼10 times lower, however, H-1152 also inhibits CaMKII, PKA, PKC, and PKG, but with IC_50_ values > 30 times higher than that for Rho-kinase (Tamura et al., 2005). H-1152 also inhibits MLCK, but with an IC_50_ value greater than 1000 times that for Rho-kinase (Tamura et al., 2005). Bis (also known as GF 109203x), besides inhibiting PKC, also inhibits PKA, PKG, and tyrosine kinase, but with values of IC_50_ 10 to 30 times greater than that for PKC (Toullec et al., 1991). The MAP kinase kinase inhibitor (U0126) appears to be rather specific, but it also inhibits PKC and extracellular signal-related kinase (ERK1/2), although with IC_50_ 150-750 times higher than that for MAP kinase kinase (Duncia et al., 1998; Favata et al., 1998).

### Inhibition of MLC Kinase Increased MAP Kinase

Inhibition of PKC with Bis or MLC kinase with ML-7 significantly increased MAP kinase phosphorylation. The Bis effect has been previously observed in our laboratory (Trappanese, 2012). The significant increase in MAP kinase phosphorylation following inhibition of MLCK was a novel finding. It suggests that MLCK may have an inhibitory effect on MAP kinase activity. It is also possible that this is due to some unknown enhancing effect of ML-7 on MAP kinase kinase. It is also noticed that nifedipine inhibited MLC phosphorylation (presumably through inhibition of MLCK) (**Figure 4**) but failed to enhance MAP kinase phosphorylation (**Figure 5**). This could be explained by assuming that MAP kinase phosphorylation is calcium dependent.

### Inhibition of MAP Kinase Kinase Increased Force

The enhancement of force during inhibition of MAP kinase kinase with U0126 was unexpected given that there was no change in the levels of caldesmon phosphorylation. Based on previous studies, U0126 should inhibit not only MAP kinase kinase but also caldesmon phosphorylation (Adam and Hathaway, 1993). MAP kinase regulates the thin filament-associated protein caldesmon by phosphorylation at Ser^789^ relieving caldesmon’s inhibitory effect on actin-activated myosin ATPase activity (Childs and Mak, 1993). MAP kinase-catalyzed caldesmon phosphorylation can be activated by stretch (Franklin et al., 1997) or contractile agonists such as KCl or phenylephrine (Dessy et al., 1998). This study suggests that MAP kinase-catalyzed caldesmon phosphorylation is not involved in the signaling pathways activated in response to opening of LTCC. Since inhibition of MAP kinase kinase increased force a possible interpretation is that activated MAP kinase may have an indirect inhibitory effect on force.

### Inhibition of ROCK Decreased Force But Not MLC Phosphorylation

ROCK has been shown to phosphorylate the regulatory MLC at Ser^19^ (Kimura et al., 1996). This function, coupled with its inhibition of MLC phosphatase activity, promotes contraction in VSM. It has been demonstrated that ROCK is overactive in VSM of hypertensive animal models and hypertensive humans, and inhibiting ROCK results in normalization of blood pressure (Uehata et al., 1997). Because ROCK plays an important role in VSM contraction its role in LTCC-induced tone was of interest. Inhibition of ROCK almost completely abolished Bay K8644-induced force. However, inhibition of ROCK did not significantly reduce MLC phosphorylation levels providing evidence for an uncoupling of force and MLC phosphorylation. Additionally, inhibition of ROCK decreased MYPT1 phosphorylation that would remove the MYPT1-dependent inhibition of MLC phosphatase activity and presumably result in a decrease in MLC phosphorylation levels.

### Bay K8644 Decreased MYPT1 Phosphorylation

MYPT1 phosphorylation significantly decreased in response to Bay K8644 stimulation. None of the kinase inhibitors, except for the ROCK inhibitor H-1152, altered the Bay K8644-dependent decrease in MYPT1 phosphorylation. Bay K8644-dependent decrease in MYPT1 phosphorylation was the opposite of what was expected to occur. Because MYPT1 phosphorylation inhibits MLC phosphatase activity it is logical to assume that addition of a contractile agonist would increase MYPT1 phosphorylation in order to enhance force production without additional Ca^2+^ influx. The only case in which MYPT1 phosphorylation was not significantly decreased was in the presence of the LTCC inhibitor nifedipine. This suggests that MYPT1 dephosphorylation may be catalyzed via a Ca^2+^-dependent mechanism. How exactly MYPT1 is dephosphorylated is unclear. Perhaps the sustained opening of LTCC caused by Bay K8644 “over-sensitized” the Ca^2+^ sensitization mechanisms and MYPT1 is dephosphorylated as a protective measure against high levels of force production.

### CPI-17 and Caldesmon Phosphorylation

In swine VSM PKCα and PKCδ are the dominant kinase for CPI-17 phosphorylation (Eto et al., 2001). Bis (or GF109203x) is a non-selective inhibitor of PKC, it inhibits the α, βI, βII, γ, δ, and ε (Gekeler et al., 1996; Alessi, 1997). Activation of PKC, phosphorylation of CPI-17, and subsequent inhibition of MLC phosphatase is part of the Ca^2+^ sensitization pathway that promotes increased MLC phosphorylation without requiring an increase in Ca^2+^ influx or release (Li et al., 1998; Throckmorton et al., 1998). The fact that CPI-17 was not phosphorylated in response to Bay K8644 is surprising because PKC activation is a common phenomenon in VSM contraction as evidenced by the fact that phorbol dibutyrate increases force in most VSM including the swine carotid artery (Sato et al., 1992; Fulginiti et al., 1993).

Caldesmon phosphorylation did not change during Bay K8644 stimulation or with the inclusion of inhibitors. The putative endogenous kinase for caldesmon is MAP kinase (Childs and Mak, 1993). Upon phosphorylation, the inhibitory effect exerted by caldesmon on actin-myosin ATPase is relieved, allowing for an increase in cross bridge cycling. We expected an increase in caldesmon Ser^789^ phosphorylation in response to Bay K8644 stimulation, and inhibition of this increase in caldesmon phosphorylation in the presence of U0126. The lack of change, increase or decrease, in caldesmon phosphorylation rules out a role for at least this thin filament protein in the regulation of vascular tone in response to opening of LTCC.

### Limitations of the Study

The study relied on the use of pharmacological inhibitors. Interpretations of the results therefore are bound by the assumption that the inhibitors are highly specific, which, as mentioned above, is not a strictly valid assumption. Findings from the study, however, could serve as clues for further investigation. Another limitation of the study is that we only focused on the pathways related to the regulation of contraction through actomyosin interaction. During the prolonged (120 min) contraction induced by Bay K8644, other pathways could have come into play, notably the pathways related to the regulation of actin cytoskeletal function and the muscle’s ability to maintain force through non-cross-bridge mechanisms (Lehman and Morgan, 2012; Yamin and Morgan, 2012; Lan et al., 2015). Both intracellular calcium and MLC phosphorylation levels are known to vary with time during contraction (Dillon et al., 1981). Many of the calcium-dependent kinase activities are therefore likely to vary during a prolonged contraction. Because we only made measurements of kinase and protein phosphorylation at one time point (120 min after stimulation), correlation of muscle force and the levels of phosphorylation of the various protein and enzymes only provides a snapshot of a complex and dynamic process.

### Overall Interpretation of Data and Summary

In this study we have shown that opening of LTCC in response to 100 nM Bay K8644 produced a sub-maximal sustained contraction that was nifedipine-sensitive. Taken together our data suggest that Ca^2+^ entry through LTCC activates the MLC kinase resulting in MLC phosphorylation and force. In addition a constitutively active ROCK and potentially ROCK that is activated by Ca^2+^ influx via LTCC (Ureña and Lopez-Barneo, 2012; Ouyang et al., 2014), sustains force by a MLC phosphatase- and MLC phosphorylation-independent mechanism. MYPT1 dephosphorylation may be Ca^2+^-dependent. PKC catalyzed CPI-17 phosphorylation and the resultant inhibition of MLC phosphatase does not occur in response to LTCC opening by Bay K8644. Inhibition of MLCK increases MAP kinase phosphorylation suggesting that MLCK may have an inhibitory or suppressive effect on MAP kinase activity. Moreover, inhibition of MAP kinase enhances Bay K8644-dependent force. Although caldesmon phosphorylation has been shown to be increased by both membrane depolarization and agonist activation, stimulation with Bay K8644 does not increase caldesmon phosphorylation. These novel findings, especially the relationship between MLCK and MAP kinase, if confirmed, may lead to a better understanding of VSM function.

## Author Contributions

HE, RM: Made substantial contributions to the conception and design of the work, the acquisition, analysis, abd interpretation of data for the work. HE, RM: Drafted the work and revised it critically for important intellectual content. CS: Made substantial contribution to interpretation of data and revising the manuscript. HE, CS: Provided final approval of the version to be published. HE, CS: Agree to be accountable for all aspects of the work and ensure that questions related to the accuracy or integrity of any part of the work can be appropriately investigated and resolved.

## Conflict of Interest Statement

The authors declare that the research was conducted in the absence of any commercial or financial relationships that could be construed as a potential conflict of interest.

The handling Editor declared a shared affiliation, though no other collaboration, with one of the authors CYS and states that the process nevertheless met the standards of a fair and objective review.
