# Corrigendum: Sustained Contraction in Vascular Smooth Muscle by Activation of L-type Ca^2+^ Channels Does Not Involve Ca^2+^ Sensitization or Caldesmon

**DOI:** 10.3389/fphar.2017.00112

**Published:** 2017-03-07

**Authors:** Hillevi K. Ets, Chun Y. Seow, Robert S. Moreland

**Affiliations:** ^1^Department of Pharmacology and Physiology, Drexel University College of MedicinePhiladelphia, PA, USA; ^2^Department of Pathology and Laboratory Medicine, University of British ColumbiaVancouver, BC, Canada; ^3^Department of Pathology and Laboratory Medicine, Drexel University College of MedicinePhiladelphia, PA, USA

**Keywords:** carotid artery, Bay K8644, nifedipine, myosin light chain kinase, protein kinase C, MAP kinase, Rho kinase

Results from our recent publication indicate that sustained contraction in vascular smooth muscle induced by Bay K8644 is independent from Rho-kinase (ROCK) associated calcium sensitization. We have failed to recognize and properly acknowledge an earlier study by Alvarez et al. (2010) that reached the same conclusion. In their study Alvarez et al. clearly demonstrated that phosphorylation of MYPT1, a downstream substrate of ROCK, was not increased by Bay K8644 activation and therefore the calcium-dependent myosin light chain (MLC) phosphorylation and the associated sustained tension maintenance cannot be the consequence of Bay K8644-induced MYPT1 phosphorylation. In our study Bay K8644 also failed to increase MYPT1 phosphorylation, and in addition we found that phosphorylation of CPI-17, a downstream substrate for PKC and ROCK, was not changed by Bay K8644 activation either. Taken together, results from both studies strongly suggest that an increase in intracellular calcium concentration in vascular smooth muscle does not necessarily lead to activation of ROCK and the associated calcium sensitization.

The concept of calcium sensitization in smooth muscle activation was first described by Morgan and Morgan (1984). In vascular smooth muscle they found that agonist (phenylephrine) stimulation, compared with the stimulation by potassium depolarization, led to more force generation at lower intracellular calcium concentrations. Rembold and Murphy (1988) also observed a stronger calcium-sensitizing effect of agonist stimulation relative to potassium depolarization. Furthermore they revealed that the sensitization step occurred in between the release of calcium and MLC phosphorylation, and not between MLC phosphorylation and force generation.

The lack of calcium-sensitizing effect of Bay K8644 has also been recognized by Rembold (1990). An important revelation of the study is that calcium sensitization is linked to stimulation of G proteins, and potassium depolarization and Bay K8644 are poor activators of G protein-coupled receptors, at least in vascular smooth muscle.

Although our report is not meant to be a review, we regret that the above cited studies were not included. We believe that addition of these references will provide our readers with a more complete history in the search for mechanisms underlying calcium sensitization.

## Conflict of interest statement

The authors declare that the research was conducted in the absence of any commercial or financial relationships that could be construed as a potential conflict of interest.
